# Genomic diversity and adaptation in Arctic marine bacteria

**DOI:** 10.1128/mbio.01555-25

**Published:** 2025-08-18

**Authors:** Michael C. Sadler, Matthias Wietz, Sayaka Mino, Robert M. Morris

**Affiliations:** 1 School of Oceanography, University of Washington 7284, Seattle, USA; 2 Deep-Sea Ecology and Technology, Alfred Wegener Institute Helmholtz Centre for Polar and Marine Research 84597 , Bremerhaven, Germany; 3 Max Planck Institute for Marine Microbiology 28267 , Bremen, Germany; 4 Institute for Chemistry and Biology of the Marine Environment, University of Oldenburg 11233, Oldenburg, Germany; 5 Laboratory of Microbiology, Faculty of Fisheries Science, Hokkaido University 204958, Hakodate, Japan; Oregon State University, Corvallis, Oregon, USA

**Keywords:** SAR11, SUP05, horizontal gene transfer, pangenomics, nanopore, Arctic, cultivation

## Abstract

Arctic marine bacteria experience seasonal changes in temperature, salinity, light, and sea ice cover. Time-series and metagenomic studies have identified spatiotemporal patterns in Arctic microbial communities, but a lack of complete genomes has limited efforts to identify the extent of genomic diversity in Arctic populations. We cultured and sequenced the complete genomes of 34 Arctic marine bacteria to identify patterns of gene gain, loss, and rearrangement that structure genomes and underlie adaptations to Arctic conditions. We found that the most abundant lineage in the Arctic (SAR11) is comprised of diverse species and subspecies, each encoding 50–150 unique genes. Half of the 16 SAR11 genomes harbor a genomic island with the potential to enhance survival in the Arctic by utilizing the osmoprotectant and potential methyl donor glycine betaine. We also cultured and sequenced four species representing an uncultured family of *Pseudomonadales*, four subspecies of *Pseudothioglobus* (SUP05), a genus of high GC *Puniceispirillales* (SAR116), and a family of low GC SAR116. Time-series 16S rRNA amplicon data indicate that this culture collection represents up to 60% of the marine bacterial community in Arctic waters. Their genomes provide insights into the evolutionary processes that underlie bacterial diversity and adaptation to Arctic waters.

**IMPORTANCE:**

Genetic diversity has limited efforts to assemble and compare whole genomes from natural populations of marine bacteria. We developed a cultivation-based population genomics approach to culture and sequence the complete genomes of bacteria from the Arctic Ocean. Cultures and closed genomes obtained in this study represent previously uncultured families, genera, and species from the most abundant lineages of bacteria in the Arctic. We report patterns of gene gain, loss, rearrangement, and adaptation in the dominant lineage (SAR11), as well as the size, composition, and structure of genomes from several other groups of marine bacteria. This work demonstrates the potential for cultivation-based high-throughput genomics to enhance understanding of the processes underlying genomic diversity and adaptation.

## INTRODUCTION

The oceans are dominated by relatively few but highly diverse lineages of marine bacteria (1, 2). Representatives for some of these lineages have been cultured from the Atlantic and Pacific Oceans, including *Pelagibacterales* (SAR11) (3, 4), *Puniceispirillales* (SAR116) (5), and *Thioglobaceae* (SUP05) (6, 7), but there is significant and unexplored genetic diversity within populations and across oceans. Metagenome-assembled genomes (MAGs) (8–10) and single-cell amplified genomes (SAGs) (11–13) have advanced the understanding of this diversity; however, MAGs and SAGs are typically incomplete and do not provide a full picture of the genetic variation within a highly diverse population of species and strains (14). Advances in cultivation have resulted in the recovery of abundant and novel marine bacteria (15), and modern sequencing techniques produce highly accurate closed bacterial genomes (16, 17). Combined, these approaches can provide more complete information about the genetic variation within bacterial populations.

The Arctic Ocean is a highly dynamic system characterized by strong spatial and temporal variation, including sea ice cover, daylight, stratification, and freshwater input (18–20). Microbes have evolved specific adaptations to survive in such extremes, such as the ability to use the compatible solute glycine betaine (21), which can enhance osmoregulation and survival in sea ice (22, 23) or can serve as a methyl donor and source of glycine (24). Some Arctic bacteria have been isolated in culture, including *Colwellia* and *Polaribacter* (25–27). However, cultivation-independent methods suggest that current Arctic culture collections represent a relatively small fraction of the community (28). Understanding the genetic variation within populations of Arctic bacteria is particularly important because the Arctic is warming at four times the global rate (29), rapidly losing sea ice (30), and encountering Atlantic water intrusion (31). These changes have the potential to impact bacterial and eukaryotic microbial diversity (32, 33), ultimately changing ecosystem functioning and biogeochemical cycles.

We conducted a high-throughput cultivation-based genomics study to gain insights into the diversity within Arctic bacterial populations. We focused on highly abundant and diverse taxa with few cultured and sequenced representatives, such as SAR11 (3, 34). SAR11 accounts for approximately 25% of bacteria in the Arctic Ocean (33, 35, 36) and is also present in sea ice (37). Like many other marine bacterial lineages, SAR11 has been classified into subclades based on 16S rRNA and internal transcribed spacer sequence analysis (38–40). These classifications are often used to identify patterns of diversity, including in the Arctic (41–43). SAR11 genomes are among the smallest for free-living bacteria (44) and have a high proportion of core genes (39), with most unique genes co-located in a ~50 Kb hypervariable region (HVR) termed HVR2 (45). The evolutionary mechanisms that maintain diversity in HVRs are poorly understood, though homologous recombination is widespread in SAR11 (46) and has been proposed as a driver of diversity in marine bacteria (47). These features have hindered efforts to resolve genetic variation and recover unique genes within populations of SAR11 (14).

This study used comparative genomics to identify patterns of gene gain, loss, and rearrangement in 16 Arctic SAR11. Several other important strains of marine bacteria representing previously uncultured species, genera, and families were also cultured and sequenced (named herein). Rationale for names assigned to previously uncultured families and genera is summarized below and detailed in the protologue. Briefly, *Candidatus* Njordibacter, the genus name derived from “Njord” (the Norse god of wind and seas), and the family *Njordibacteraceae* to encompass this genus. *Candidatus* Levibacter, the genus name derived from the Latin “levis” (lightweight), and the family *Levibacteraceae* to encompass this low GC genus of *Puniceispirillales. Candidatus* Ponderosibacter, the genus name derived from the Latin “ponderosus” (heavy), referring to this genus of the high GC *Puniceispirillales. Candidatus* Marifrigoribacter, the genus name derived from the Latin “mare” (of the sea) and “frigus” (cold). All 34 new species names correspond to their cultivation ID (e.g., sp. uisw_002).

## RESULTS

### High-throughput cultivation-based genomics

**Fig 1:**
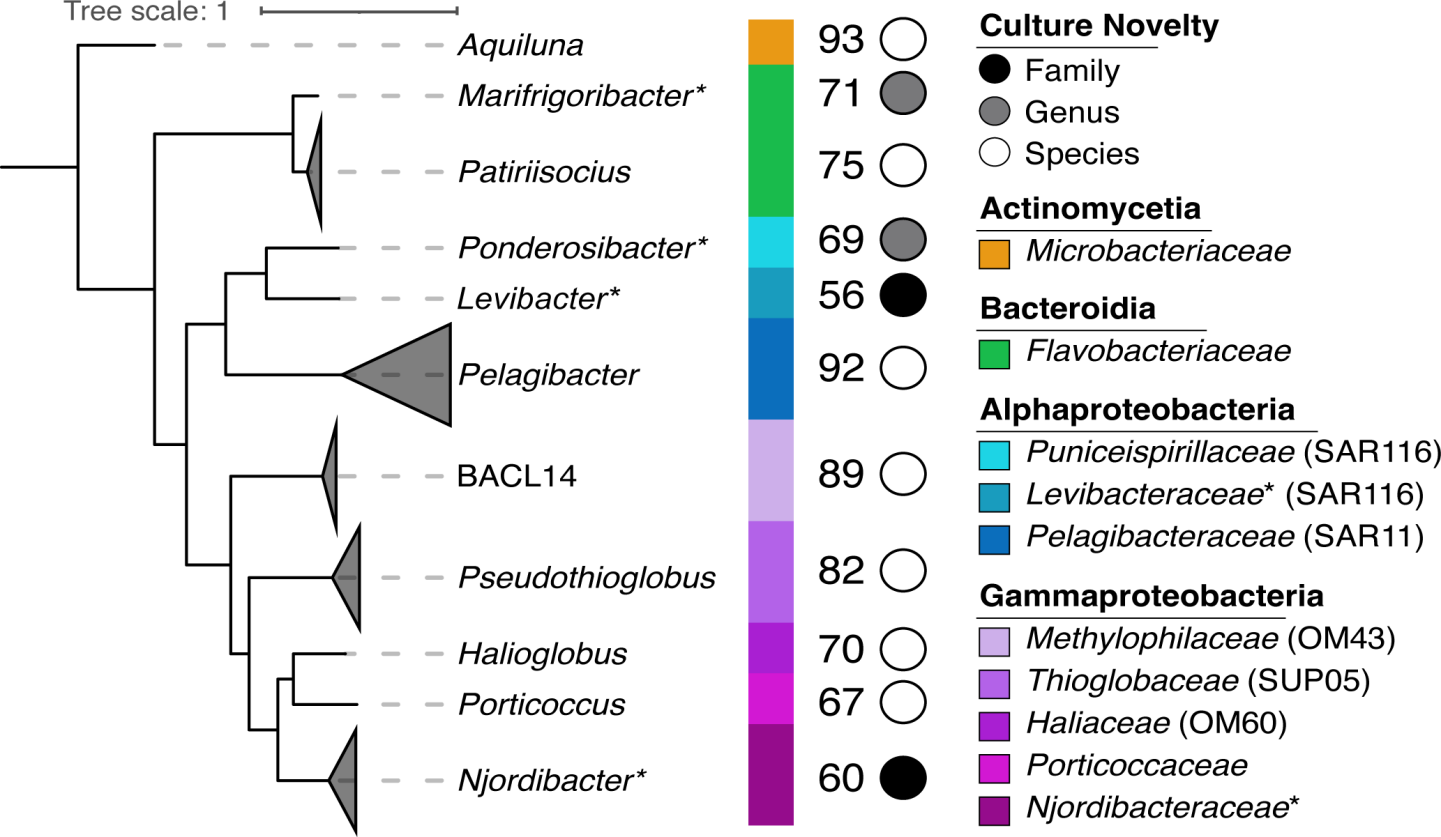
Diversity of Arctic bacteria cultured and sequenced herein. Phylogenomic analysis of Arctic marine bacteria sequenced in this study, with genus affiliation (italics), class and family taxonomic grouping (color bars), maximum amino acid identity (AAI) of all branch members to the closest cultured relative (number), and culture novelty in circles (family, genus, and species). Wedges are proportional to the number of cultures sequenced for each lineage. Family and genus names were assigned using the Genome Taxonomy Database (GTDB)-Tk placement tool, except for those proposed in this study, marked with an asterisk.

We cultured and sequenced the complete genomes of 34 Arctic marine bacteria to identify differences in genomic diversity and adaptations to Arctic conditions (Fig. 1). Cultures were selected from 106 bacteria obtained by high-throughput dilution-to-extinction cultivation and sequenced using the Oxford Nanopore Technologies (ONT) platform (Table S1). All genomes are single circular contigs, range in size from 1.29 to 3.78 Mbp, and have GC contents between 29% and 52% (Table S2). They include representatives from globally distributed lineages, such as *Pelagibacter* (SAR11, *n* = 16), *Pseudothioglobus* (SUP05, *n* = 4), *Puniceispirillales* (SAR116, *n* = 2), *Methylophilaceae* (OM43, *n* = 2), and *Haliaceae* (OM60, *n* = 1), as well as uncultured and understudied lineages, including a low GC family of SAR116 (48) and a previously unrecognized family of *Pseudomonadales* that is common in the Arctic (*Njordibacteraceae* herein). Other cultured strains belonging to genera and species without previous representation were also sequenced, including *Flavobacteriaceae* and *Actinomycetes*.

All genomes represent newly cultured species (Fig. 1, Table S1). The four SUP05 genomes have 95% average nucleotide identity (ANI) when compared to each other and less than 95% ANI when compared to described species. The SUP05 genomes encode key proteins needed to fix inorganic carbon using the Calvin-Benson-Bassham cycle, including phosphoribulokinase and the large and small subunits of form Ia Ribulose Bisphosphate Carboxylase. *Levibacter* and *Ponderosibacter,* belonging to the SAR116 clade, have GC contents of 31.4% and 49.8%, coding densities of 95.9% and 88.5%, and amino acid identity (AAI) of 56% and 69% when compared to their nearest cultured relatives, respectively (Table S2). Phylogenetic analysis indicates these cultures represent distinct and diverse SAR116 lineages (Fig. S1). Both SAR116 genomes encode genes for bacteriorhodopsin, carotenoid biosynthesis, carbon monoxide dehydrogenase, and key proteins for sulfur metabolism. The *Porticoccus, Patiriisocius,* and *Halioglobus* genomes have low AAI values when compared to their nearest cultured relatives (67%, 75%, and 70%, respectively), indicating substantial diversity in these lineages. The *Marifrigoribacter* genome has a similarly low AAI value when compared to its nearest cultured relative (71%) and represents the first genome of an uncultured genus of *Flavobacteriaceae*. Notably, the *Porticoccus* genome has 67% AAI and is 40% (1.4 Mbp) smaller than its nearest cultured relative.

### Spatial and temporal abundance of Arctic bacteria

**Fig 2:**
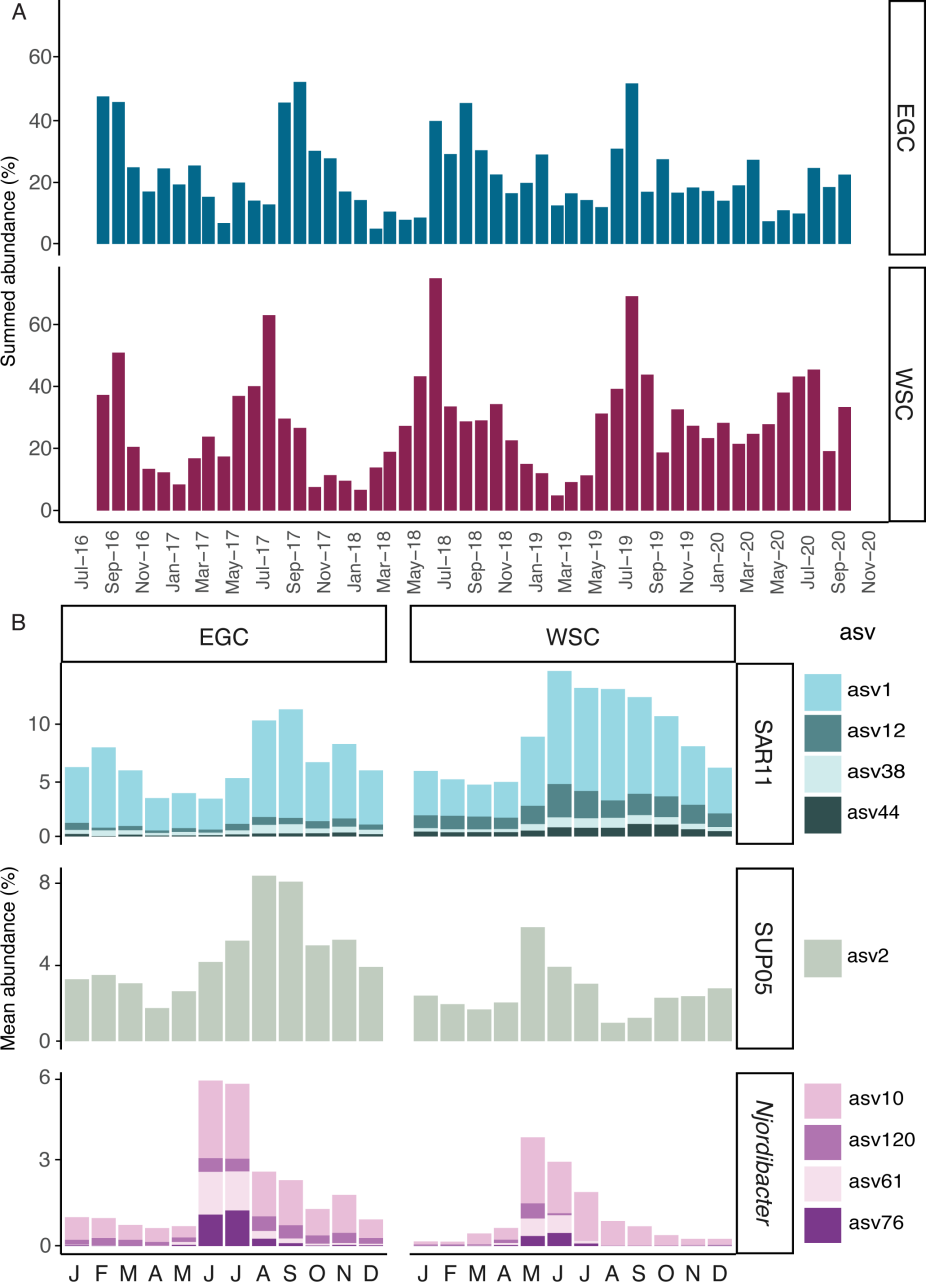
Relative abundance of bacterial ASVs from the FRAM Observatory with 100% identity to 16S rRNA gene sequences from cultures obtained in this study. (**A**) Summed relative abundance of all matching ASVs from August 2016 to September 2020 in the EGC and WSC. (**B**) Mean relative abundance values calculated for ASVs matching sequenced *Pelagibacter* (SAR11), *Pseudothioglobus* (SUP05), and *Njordibacter* cultures in the EGC (left) and WSC (right). Bars in panel B represent monthly averages. Individual ASVs for each lineage in panel B are shown on the right.

Spatiotemporal abundance estimates for bacteria cultured in this study were determined by aligning the 16S rRNA gene sequences from cultures with 16S rRNA amplicon sequence variants (ASVs) from two continuous time-series (Fig. 2). These 4-year Arctic datasets were obtained from the West Spitsbergen Current (WSC) and the East Greenland Current (EGC) (49, 50). The 34 cultures matched 18 ASVs with 100% sequence identity (Table S2) and constituted over 60% of the ASV abundance in the WSC and 50% of the ASV abundance in the EGC (Fig. 2A). The most abundant members in the culture collection included SAR11, SUP05, and *Njordibacter*, accounting for 4%–15% of the community in the WSC and 6%–10% of the community in the EGC at peak abundances, respectively (Fig. 2B). ASVs matching SUP05, *Njordibacter,* and *Marifrigoribacter* cultures were more abundant in the EGC, while those matching SAR11, SAR116, and OM43 cultures were more abundant in the WSC (Fig. 2; Fig. S2). Spearman correlation analyses indicate that many of the ASVs matching cultured taxa covary with environmental parameters measured over the same 4 years (Fig. S3). ASVs matching SAR11, SAR116, and OM43 cultures correlate positively with water temperature and negatively with polar water fraction, while ASVs matching *Njordibacter* and SUP05 cultures correlate negatively with water temperature and positively with polar water fraction.

A relatively small fraction of the total SAR11, SUP05, and *Njordibacter* ASVs matched sequences in cultures (4/116, 1/55, and 4/24, respectively). However, these nine ASVs accounted for a substantial percentage of total SAR11, SUP05, and *Njordibacter* ASV abundance detected in the Arctic time-series (70%, 50%, and 90%, respectively) (Fig. S4). Differences in genome and ASV diversity are notable between the groups (Fig. S5; Tables S2 and S3). Many SAR11 genomes are unique species (<95% ANI) but match the same ASV (e.g., uisw_099_02 and uisw_114 matching ASV38), while SAR11 uisw_121 and uisw_116 are the same species (>95% ANI) but match different ASVs. Similarly, *Njordibacter* uisw_002 and uisw_058 have 80% ANI to each other and match the same ASV, while different copies of the 16S rRNA gene in *Njordibacter* uisw_056 match different ASVs. In contrast, all SUP05 genomes represent the same species (>95% ANI) and match the same ASV.

### Gene gain, loss, and rearrangement in Arctic SAR11

**Fig 3:**
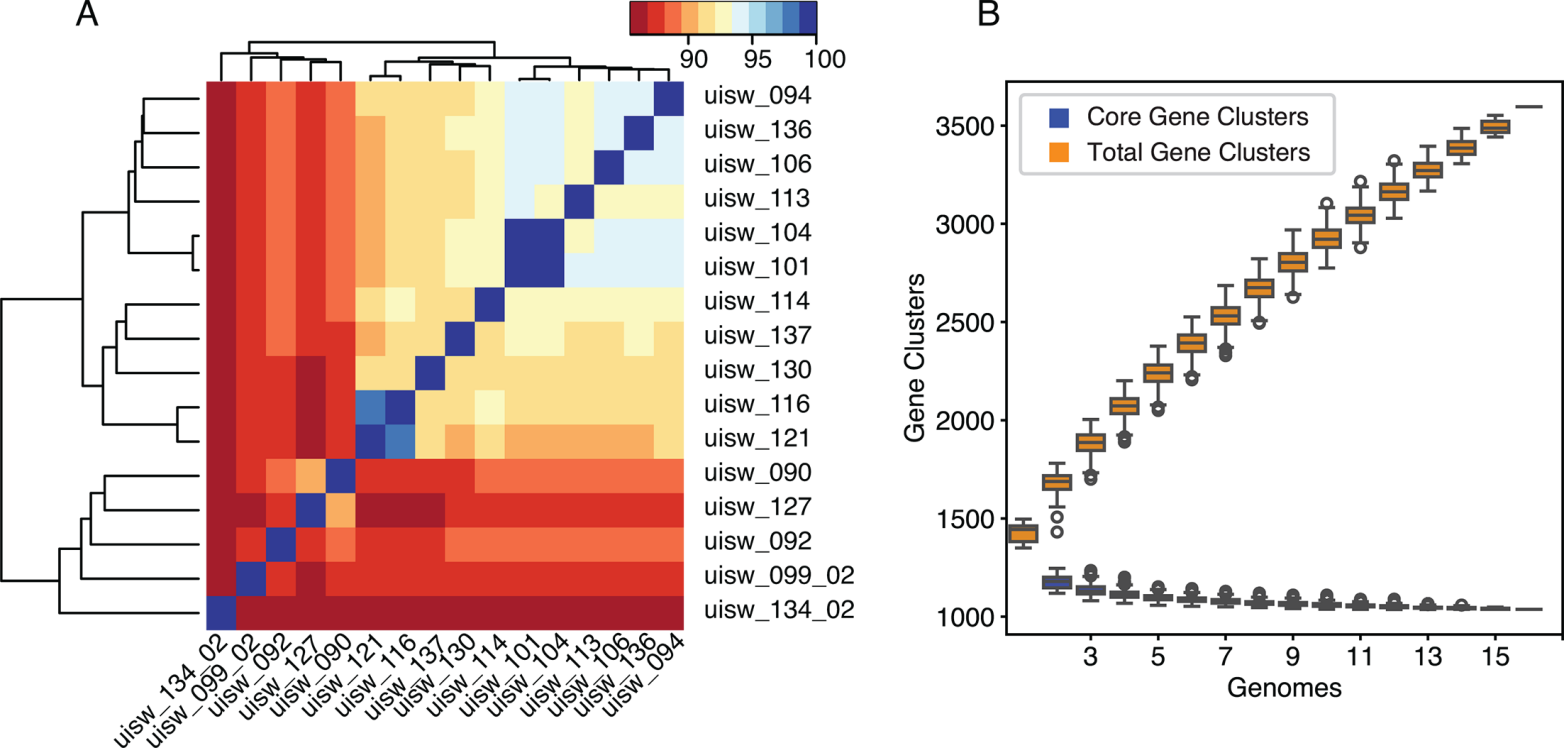
Genomic diversity of Arctic SAR11 cultures. (**A**) Clustered ANI for each pairwise genome comparison. (**B**) Gene cluster accumulation curves for the Arctic SAR11 population, total gene clusters (orange), and core gene clusters (blue). Accumulation curves were calculated using 1,000 iterations.

We evaluated the diversity of SAR11 genomes, which are abundant in the Arctic and well represented in our culture collection, to identify population-wide patterns of genomic diversity. All 16 Arctic SAR11 cultures obtained in this study are members of subclade Ia.1 (Fig. S6). Twelve have ANI values that are <95% to each other, and two pairs have ANI values >95% to each other, indicating that they represent 14 new species and two new subspecies of SAR11 (Fig. 3A; Table S3). Population genomic analysis identified 3,596 total gene clusters, 1,661 singleton gene clusters, and 1,037 core gene clusters (Fig. 3B). Each genome encodes between 1,385 and 1,477 genes. The number of shared genes between any two genomes ranges from 1,126 to 1,234, and the number of singletons in each genome ranges from 50 to 150.

**Fig 4:**
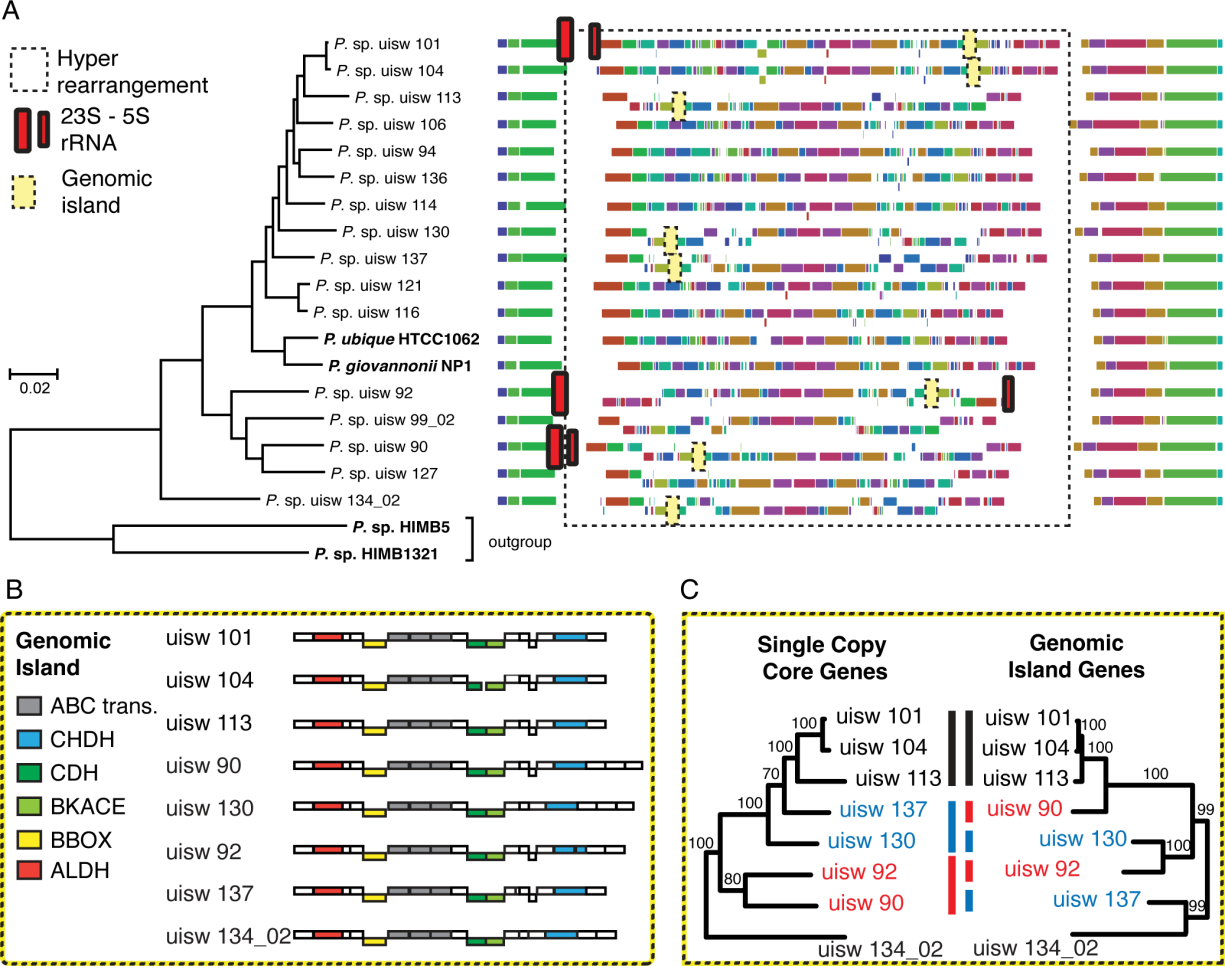
Structure of Arctic SAR11 genomes. (**A**) Phylogenomic analysis and whole-genome alignment of Arctic SAR11. (**B**) Alignment and gene content of a genomic island identified in eight SAR11 genomes. (**C**) Comparison of phylogenetic trees constructed from concatenated protein sequences of single-copy core genes (left) and genomic island genes (right). Phylogenetic trees were constructed with translated and concatenated sequences. Whole genome alignments start with DnaA. In all genomes, the relative position of the 23S and 5S rRNA genes is as depicted for uisw_101, unless otherwise indicated. Previously sequenced isolates are in bold. Enzyme names in panel B are abbreviated as follows: ABC *Trans,* ABC transporter; CHDH, choline dehydrogenase; CDH, carnitine dehydrogenase; BKACE, beta-keto acid cleavage enzyme; BBOX, gamma-butyrobetaine dioxygenase; ALDH, aldehyde dehydrogenase. Bootstrap values in panel C are displayed at each node. Black, red, and blue color blocks highlight node rearrangements.

A whole genome alignment with all 16 Arctic SAR11 and two closely related reference genomes (*Pelagibacter ubique and Pelagibacter giovannonii*) was constructed to identify patterns of gene gain, loss, and rearrangement. The alignment revealed notable patterns of rearrangement (Fig. 4). Most notably, there is an ~880 Kb section bound by the 23S and 5S rRNA genes in uisw_092 (Fig. 4A). Patterns of gene gain, loss, and rearrangement in this region were used to identify a unique genomic region that has 17–20 genes and is present in half of the sequenced genomes (Fig. 4B; Table S4). Genes in this region code for a glycine betaine ABC transport system (ABC *trans*.), a choline dehydrogenase (CHDH), an aldehyde dehydrogenase (ALDH), a carnitine dehydrogenase (CDH), a gamma-butyrobetaine dioxygenase (BBOX), and a beta-keto acid cleavage enzyme (BKACE). Gene order for these genes is preserved and always upstream of genomic betaine/choline/proline ABC transport genes. Phylogenetic trees constructed using concatenated protein sequences from these genes and single-copy core genes are incongruent (Fig. 4C). Gene cluster analysis indicates that of the 17 genes, 15 are unique to the region, with no identifiable homologs occurring elsewhere in the genomes. The remaining two form phylogenetic clusters that are distinct from clusters produced using other copies found in the genomes, such as for aldehyde and choline dehydrogenases (Fig. S7A and B). Most genes in this region (13 out of 17) produced phylogenies that were congruent with each other and with the concatenated set of all 17 genes, including the aldehyde and choline dehydrogenase genes found in this region (Fig. S7C and D).

**Fig 5:**
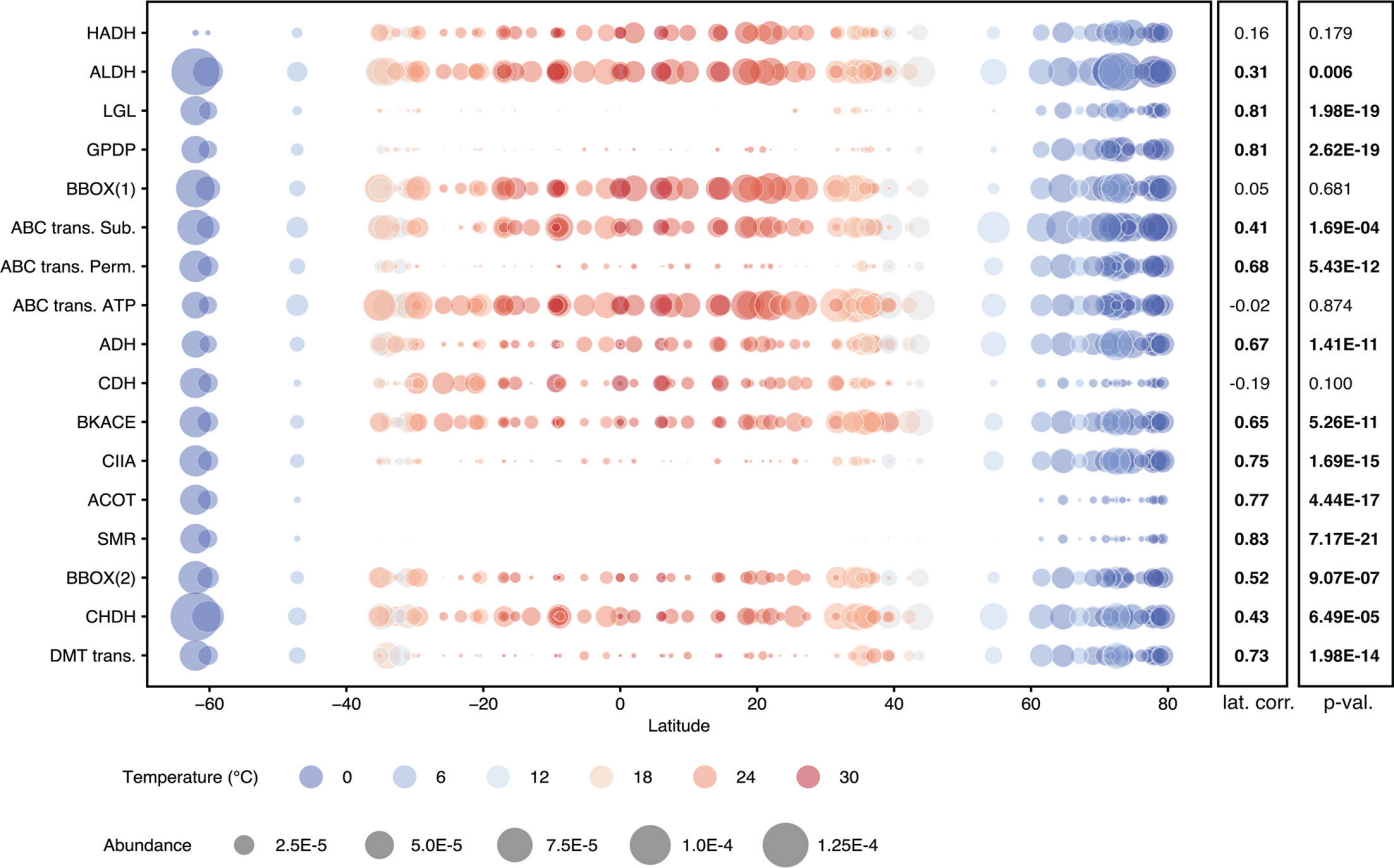
Latitudinal distribution of genes identified in an Arctic SAR11 genomic island. Representative nucleotide sequences from uisw_090 were used to identify homologs in the Ocean Gene Atlas OMRGC v2 metaG data set for all 17 island genes (Table S4). The Spearman’s correlation (lat. corr.) and *P* value (p-val.) between abundance and latitude are displayed to the right for each gene. Enzyme names are abbreviated as follows: HADH, 3-hydroxyisobutyrate dehydrogenase; ALDH, aldehyde dehydrogenase; LGL, lactoylglutathione lyase; GPDP, glycerophosphoryl diester phosphodiesterase; BBOX(1), gamma-butyrobetaine dioxygenase; ABC *trans*. Sub, ABC transporter substrate binding; ABC *trans*. Perm., ABC transporter permease; ABC *trans*. ATP, ABC transporter ATP binding; ADH, alcohol dehydrogenase; CDH, carnitine dehydrogenase; BKACE, beta-keto acid cleavage enzyme; CIIA, Class II aldolase; ACOT, acyl-CoA thioesterase; SMR, small multidrug resistance transporter; BBOX(2), gamma-butyrobetaine dioxygenase; CHDH, choline dehydrogenase; DMT trans., drug/metabolite transporter.

We searched for this genomic region in public databases to see if it was common but previously unrecognized. There was no evidence of this arrangement in 18 previously sequenced single-contig SAR11 genomes (Table S5). Homologs for the 17 encoded genes were identified in environmental databases. Abundances for 13 have a significant positive correlation with latitude (0.31–0.83, *P* value < 0.01) (Fig. 5), and seven are rare or absent in samples from lower latitudes, including a lactoylglutathione lyase, a glycerophosphodiester phosphodiesterase, the permease subunit of a proline/glycine betaine ABC transporter, a class II aldolase, a thioesterase, a small multidrug resistance (SMR) transporter, and a drug/metabolite transporter (DMT).

### A new family of *Pseudomonadales*

**Fig 6:**
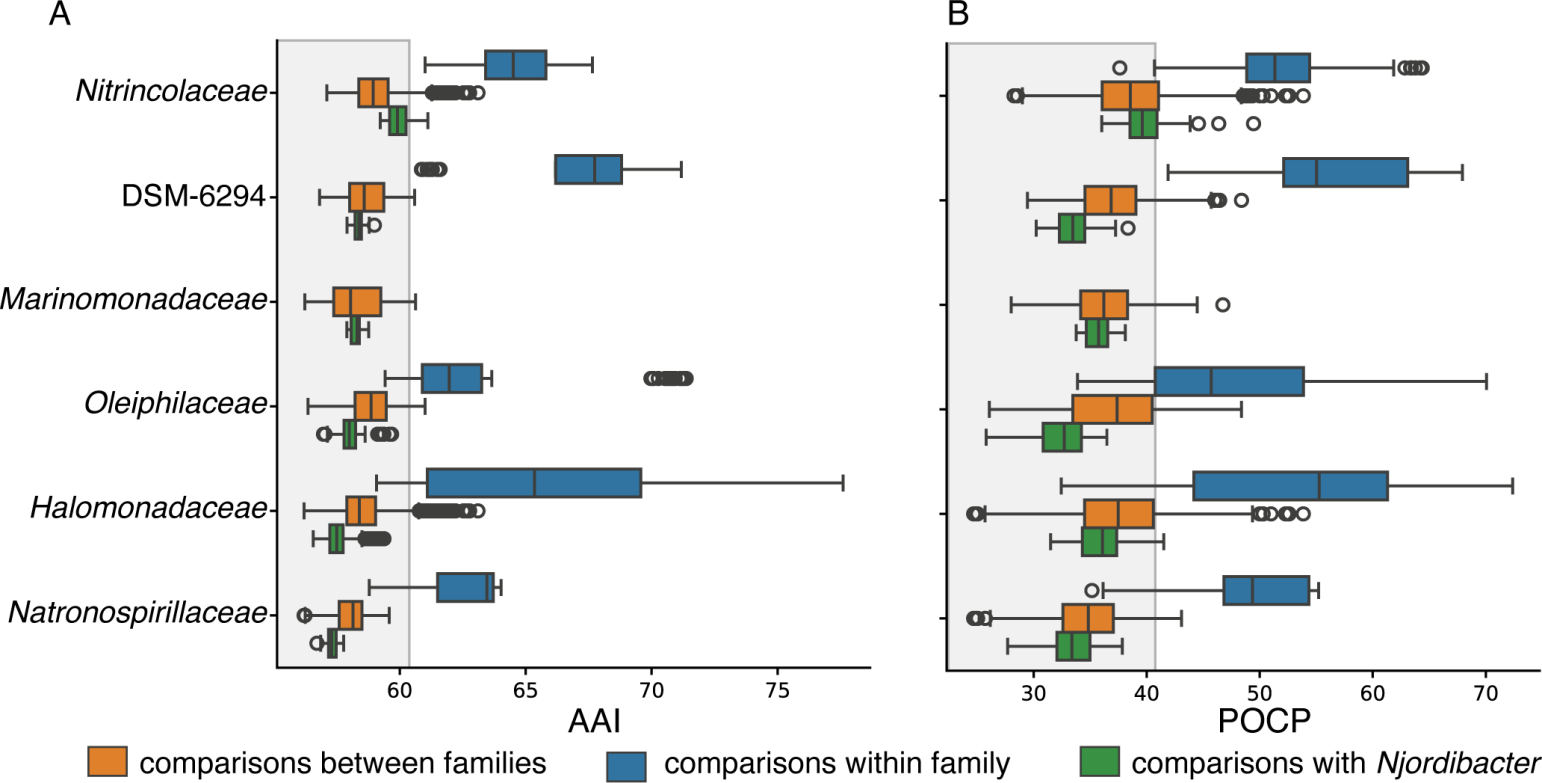
Whole-genome-based family and genus delineations in the order *Pseudomonadales*. (**A**) All pairwise average AAI comparisons. (**B**) All pairwise POCP comparisons. Representatives from the six most closely related families to *Njordibacter* were analyzed (Table S6). The shaded box indicates the family-level delineation in the order *Pseudomonadales*. Circles represent values outside the 1.5 interquartile range from the first and third quartiles.

A group of *Pseudomonadales* (named *Njordibacter* herein) was frequently identified in culture (*n* = 10). Four cultures were selected for whole genome sequencing and used to identify a new family in the order *Pseudomonadales* (Fig. 6). The genomes range from 2.4 to 2.9 Mbp in length, have a GC content of 43.8%–45.1%, and each contains four copies of the 16S rRNA gene (Table S2). Pairwise ANI values range from 79% to 82% when compared to each other. One genome, uisw_056, and a previously characterized MAG from the Arctic (49) have 99.6% ANI. Phylogenomic analysis using the Genome Taxonomy Database (GTDB) (51) placed this group in a deeply branching and uncultivated genus (ASP10-02a) of *Nitrincolaceae*, but with low AAI (60%) and 16S rRNA sequence similarity (91.7%) to the most closely related isolate. Additionally, the mean relative evolutionary divergence (RED) of this group was 0.875, falling within both the genus and family ranges (52). We therefore compared the genomes of 114 isolates in the order *Pseudomonadales* (Table S6) to each other and to the four *Njordibacter* genomes using AAI and percent of conserved protein (POCP). This yielded over 10,000 pairwise comparisons between 34 genera from seven families, from which family delineations could be distinguished (Fig. 6). Values of 60% AAI and 40% POCP with their closest *Nitrincolaceae* relatives are below the family delineation line, indicating that these *Njordibacter* genomes represent a new family in the order *Pseudomonadales*.

## DISCUSSION

### Extensive genomic diversity in Arctic SAR11

Our analysis identified 1,037 core genes in 16 SAR11 subclade Ia.1 genomes obtained from Arctic seawater (Fig. 3B). A similar estimate of 1,047 core genes was obtained in a SAR11 pangenome analysis of five complete genomes from subclade Ia, isolated from diverse locations in the Atlantic and Pacific Oceans (39). Similarity between the core genome estimates using 16 genomes from the Arctic and five genomes from different oceans highlights the extent of core genome conservation within the SAR11 clade (34, 39). The SAR11 pangenome analysis also identified 1,962 total unique gene clusters in the five complete genomes (39). The mean number of unique gene clusters in five Arctic SAR11 genomes was 2,241 ± 117 (95% CI), suggesting that there is greater gene content diversity among Arctic populations of SAR11. This could be due to the mixing of different populations from Arctic and Atlantic waters (19, 20) or due to enhanced genetic diversity within Arctic populations, as a previous study found that microbial diversity increased with latitude and decreased with temperature (53). Regardless, our analyses suggest that substantial sequencing effort is needed to estimate the gene content and total number of SAR11 species and subspecies in a SAR11 population.

A translocation of the 5S rRNA gene in SAR11 uisw_092 was used to define a large (~880 kbp) genomic region with a higher frequency of inversions and gene indels relative to regions flanking the origin of replication, suggesting that there is more selective pressure to conserve gene order near the origin of replication (Fig. 4A). We also identified a genomic region that encodes genes for the uptake and production of glycine betaine (Fig. 4; Table S4). Evidence that phylogenies for genes in this region are congruent with each other and incongruent with the phylogeny of single-copy core genes suggests that this region is a genomic island acquired by horizontal gene transfer (HGT) (Fig. 4C; Fig. S7). Although the insertion site appears to be conserved, there are no obvious genes that facilitate HGT in the region. However, homologous recombination is widespread in SAR11 (46) and has been proposed as a mechanism for the transfer of genomic islands (47). It has also been proposed as a mechanism for genomic diversification in other marine bacteria, including HIMB59 (a sister clade to SAR11) and OM43 (54, 55). In the case of HIMB59, more phosphorus-related genes were identified on a genomic island under phosphorus-limiting conditions (54). Similarly, the genomic island encoding genes for the uptake of glycine betaine in Arctic SAR11 could enhance the survival and metabolic activities of cells experiencing low temperatures and high salinities during freezing, as reported for other bacteria (21–23). The island may also serve as an important methyl donor and source of glycine (24). Our evidence that genes in the Arctic SAR11 genomic island are more abundant at polar latitudes and less abundant or absent in temperate regions suggests that it is an adaptation to Arctic conditions, maintained in a large fraction of the population (50%) by HGT (Fig. 5). A similar polar distribution pattern has been observed in metagenomic read recruitment for SAR11 clade Ia.1 (56), suggesting that this could give SAR11 clade Ia.1 populations an advantage during the annual formation and melting of sea ice.

### Variable patterns of diversity in abundant Arctic populations

Time-series ASV data from the Fram Strait suggest that the most frequently cultured bacteria in our culture collection (SAR11, SUP05, and *Njordibacter*) are among the most abundant Arctic taxa (Fig. 2B). There are, however, notable differences in the diversity of ASVs between these lineages and their matches to genomes in our culture collection (Fig. S5). Many unique species of SAR11 and *Njordibacter* match the same ASV, and in some cases, unique ASVs match the same species or different copies of the 16S rRNA gene in the same genome. These observations underline that ASVs cannot delineate patterns of bacterial speciation (57). However, closed genomes can be used to significantly improve estimates of diversity that are based on ASV analyses. Evidence that the Arctic SUP05 population is comprised of subspecies with similar genomes with relatively high ANI values also raises questions about how genomic diversity is maintained, particularly when compared to SAR11. Higher frequencies of genomic rearrangement have been reported as a mechanism for diversification in marine nitrogen fixers (58). A similar mechanism may help maintain diversity in SUP05, although more closed genomes are needed to quantify differences in the frequencies of gene gain, loss, rearrangement, and recombination in both SUP05 and SAR11.

### Sulfur metabolism in high and low GC SAR116

 The SAR116 clade consists of high GC (50% ± 7%) and low GC (31% ± 1%) lineages (48). Both clades are globally distributed, but metagenomic and single-cell analyses have suggested distinct differences in their ability to metabolize dimethylsulfoniopropionate (DMSP) (48). Specifically, high GC lineages encode DMSP lyases (*dddL* and *dddP*), which produce a volatile compound resulting in a loss of sulfur to the atmosphere as dimethyl sulfide (DMS). In contrast, low GC lineages encode DMSP demethylases (*dmdA*), which retains sulfur as 3-(methylsulfanyl)propanoate. Interestingly, the high GC SAR116 we cultured from the Arctic, *Ponderosibacter*, encodes *dmdA*, indicating that some high GC members also demethylate DMSP. This highlights the importance of closed genome sequences to verify the presence, and more importantly, the absence of key metabolic functions.

### Conclusion

Early efforts to assemble genomes of marine bacteria directly from seawater produced only partial sequences for the most abundant lineages (59). While advances in sequencing have improved our ability to identify genetic variation in nature, the extent of genomic diversity within bacterial populations remains elusive, particularly for abundant lineages such as SAR11 (10, 14). Our high-throughput cultivation-based genomics approach produced 34 closed bacterial genomes, including several for the most abundant lineages in the Arctic (Fig. 2). These cultures and genomes provide insights into the diversity and adaptation of Arctic populations, as well as high-quality reference sequences that will enable population genomic analyses when similar high-quality sequences become available for populations in other oceans.

## MATERIALS AND METHODS

### Sample collection

Seawater samples were collected in the Arctic Ocean aboard the RV Kronprins Haakon from 18 to 21 May 2023, through an auger hole bored through ~2 m thick sea ice. Samples for cultivation (50 mL) were collected at 81.04° N, 10.62° E, from 25 m below the ice/water interface with a temperature of −1.8°C, salinity of 34.2 PSU, and 0.28 relative flourescence units, using a 2 L Hydro-Bios water sampler (Hydro-Bios, Altenholz, Germany). A 1 mL seawater sample was amended to 10% (vol/vol) glycerol, flash frozen in liquid nitrogen, and stored at −80°C until high-throughput dilution-to-extinction cultivation. Seawater for culture media (10 L) was collected at 80.96° N, 9.66° E from 1 m below the ice/water interface into acid-washed and Milli-Q rinsed cubitainers. Seawater was then filter-sterilized using a tangential-flow-filtration (TFF) system equipped with a 30 kDa Pellicon XL Polyethersulfone Biomax filter (MilliporeSigma, Burlington, MA). The resulting media was collected in 1 L acid-washed and autoclaved polycarbonate bottles, incubated for 2 months at 4°C, and checked for bacterial growth with a flow cytometer to ensure sterility prior to use.

### Bacteria cultivation

Cultures were obtained by high-throughput dilution-to-extinction cultivation using cryopreserved Arctic seawater as previously described (60). Briefly, 0.059 mL of cryopreserved seawater with a cell density of 2.4 × 10^5^ cells per mL was diluted to ~33 cells per mL in 30 kDa filter-sterilized seawater media. A 1.5 mL aliquot was then added to each well of three acid-washed and sterile 96-well Teflon plates, incubated at 4°C, and monitored for growth once a week for 13 weeks using a Guava easyCyte flow cytometer (Cytek, Fremont, CA). Wells that were positive for growth (>2 × 10^4^ cells per mL) were subjected to 16S rRNA sequence analysis when cell densities reached >10^5^ cells per mL and preserved in 10% (vol/vol) glycerol that was frozen and stored at −80°C.

### Culture identification

DNA for PCR was extracted from 100 µL of cell culture and sequenced using a physical lysis procedure as previously described (61). Briefly, potassium hydroxide–dithiothreitol was added, and samples were subjected to one freeze-thaw cycle. The pH was then adjusted to 8.0 using Tris-HCl. DNA was purified using 2× (vol/vol) DNA mag beads (Sergi Lab Supplies, Seattle, WA) with two 80% ethanol washes and eluted in 20 µL 10 mM Tris-HCl. 16S rRNA gene fragments were then amplified using PCR with primers 27F_B (5′ AGRGTTYGATYMTGGCTCAG 3′) and 926R_B (5′ CCGYCAATTCMTTTRAGTTT 3′), and in some cases using a second semi-nested PCR reaction with primers 519F (5′ CAGCMGCCGCGGTAATWC 3′) and 926R_B. The following PCR conditions were used throughout: 94°C 120 s, 38 cycles of amplificaiton (94°C 20 s, 55°C 45 s, 72°C 120 s). PCR products were sequenced by Genewiz (Genewiz, Seattle, WA). Cultures were putatively identified by aligning sequences in the Silva database v138.1 (62, 63).

### Genome sequencing

Cultures selected for whole-genome sequencing were revived from freezer stocks in 1 L acid-washed and autoclaved polycarbonate bottles containing TFF sterilized Puget Sound seawater media. Cells were collected on 47 mm 0.2 µm pore size Isopore membrane filters (MilliporeSigma, Burlington, MA) when cultures reached maximum cell densities (between 10^5^ and 10^6^ cells per mL). High molecular weight DNA was extracted using the AutoGen QuickGene DNA Tissue Kit (Autogen, Holliston, MA) following the extraction protocol for animal tissue with minor modifications as noted below. Filters were cut into small pieces using sterile forceps and scissors and placed in sterile 2 mL DNA LoBind tubes (Eppendorf, Hamburg, Germany) containing 200 µL of TE buffer. Filters were then frozen at −80°C for 20 minutes and heated until thawed at 95°C. All recommended extraction volumes were doubled, and DNA was eluted in 200 µL of molecular-grade water. DNA was cleaned using 1× (vol/vol) DNA magnetic beads (Sergi Lab Supplies, Seattle, WA) with two 80% ethanol washes, then eluted in 20 µL of molecular-grade water. DNA was quantified using a Qubit dsDNA HS kit (Invitrogen, Waltham, MA) and sequenced using the ONT R10.4.1 Flongle flow cells with the SQK-RAD114 rapid library prep kit (Oxford Nanopore Technologies, Oxford, United Kingdom). Bases were called with Dorado v4.2.0 (github.com/nanoporetech/dorado), using the dna_r10.4.1_e8.2_400bps_hac@v4.2.0 model.

### Genome assembly and annotation

Bacterial genomes were assembled with Flye v2.9.1 (64) and polished with Medaka v1.7.2 (github.com/nanoporetech/medaka) using the UseGalaxy web platform (65). Genome annotation was performed by NCBI using the Prokaryotic Genome Annotation Pipeline v6.8 (66, 67). The quality of ONT genomes was evaluated by comparing ONT-only genomes constructed with varying levels of coverage (9–500×) to ONT-Illumina hybrid genome assemblies obtained for two previously sequenced strains, the SAR11 strain NP1 (68) and the SUP05 strain EF3 (69). Coverage for ONT-only genomes was varied by subsampling ONT reads (accessions: SRX26378910 and SRX22361185) with Rasusa v2.0.0 (70). Hybrid genomes were created by polishing ONT genomes with Illumina reads (accessions: SRX26378911 and SRX23025519) using BWA-mem2 v2.2.1 (71) and Pilon v1.2.0 (72). The quality of ONT-only genomes was determined by identifying the number of mismatches, indels, and excess CDSs relative to hybrid assemblies using Quast v5.2.0 (73) (Fig. S7). Only closed genomes with greater than 10× coverage, corresponding to >99.9% accuracy, were used for further analyses.

### Spatial and temporal abundance

16S rRNA sequences of cultures were matched against 5,511 ASVs derived from the HAUSGARTEN/FRAM Observatory in Fram Strait. ASVs originated from year-round autonomous sampling in the polar-influenced East Greenland Current (49) and the Atlantic-influenced West Spitsbergen Current (50) between 2016 and 2020, with approximately biweekly resolution. Using Geneious Prime v2023.2.1 and R v4.2.2, we investigated their abundances and environmental correlations in Fram Strait over time to establish a broader spatiotemporal and ecological context. The latitudinal distributions of genes within the Arctic SAR11 genomic island were determined by identifying homologs for each gene in the Ocean Gene Atlas OMRGC v2 metaG data set. Representative nucleotide sequences from uisw_090 were used in a BLAST search of the database with an expect threshold of E-10 or less. The abundance of each homolog was normalized to the percent of mapped reads, as previously described (74, 75). Homolog abundance for each gene at each station were summed. Spearman’s correlations were calculated for latitudinal distance from the equator using the “spearmanr” function in scipy v1.14.0 (76), and corrected for multiple hypothesis tests using the Benjamini/Hochberg method from statsmodels v0.14.2 (77).

### Phylogenomics and population genomics

Taxonomic classifications were assigned to sequenced genomes using the GTDB-Tk v2.3.2, with the *de novo* workflow and reference database v214 (78). New species and strains were given the prefix “uisw” followed by the cultivation number. Most genomes came from pure cultures (*n* = 27). The suffix “_01” or “_02” was added to the cultivation number if two complete genomes were recovered from a mixed culture. The genomes of these cultures were analyzed using average AAI with ezaai v1.2.3 (79), POCP with POCP v2.3.2 (80), ANI with pyANI v0.2.12 (81), and RED with PhyloRank v0.1.12 (github.com/dparks1134/PhyloRank), using the GTDB v214 database. Genome structure was evaluated through the visualization of linear co-similarity blocks using progressiveMauve v2015-02-25 (82). All phylogenetic trees were constructed with MUSCLE v3.8.31 (83) and RAxML v8.2.11 with model GTRGAMMA (84) using the ETE3 v3.1.3 phylogenetic analysis pipeline (85). Whole-genome phylogenies were constructed using the bacterial_71 single-copy core gene collection in anvi’o v8 (86). SAR11 gene cluster data were created using the anvi’o pangenomic workflow with the flags –use-ncbi-blast, –minbit 0.5, and –mcl-inflation 10, as previously described (87).

### Protologue

#### *Candidatus* Njordibacter gen. nov.

Njor.di.bac’ter. N.L. masc. n. *bacter*, rod; N.L. masc. n. *Njordibacter*, a rod named after *Njord,* the Norse god of wind and seas.

#### *Candidatus* Njordibacteraceae fam. nov.

Njor.di.bac.te.ra.ce’ae. N.L. masc. n. *Njordibacter*, type genus of the family; suff. -aceae, ending to denote a family; N.L. fem. pl. n. *Njordibacteraceae*, the family of the genus *Njordibacter*. The description of the family *Njordibacteraceae* is the same as for the genus *Njordibacter*.

#### *Candidatus* Levibacter gen. nov.

Le.vi.bac’ter. L. masc. adj. *levis*, light in weight; N.L. masc. n *bacter*, rod; N.L. masc. n. *Levibacter,* a light rod alluding to the low GC clade of the order *Puniceispirillales*.

#### *Candidatus* Levibacteraceae fam. nov.

Le.vi.bac.ter.a.ce’ae. N.L. masc. n. *Levibacter*, type genus of the family; suff. -aceae, ending to denote a family; N.L. fem. pl. n. *Levibacteraceae*, the family of the genus *Levibacter*. The description of the family *Levibacteraceae* is the same as for the genus *Levibacter*.

#### *Candidatus* Ponderosibacter gen. nov.

Pon.de.ro.si.bac’ter L. masc. adj. *ponderosus,* heavy, weighty; N.L. masc. n. *bacter*, rod; N.L. masc. n. *Ponderosibacter*, a heavy rod alluding to the high GC lineage of the order *Puniceispirillales*.

#### *Candidatus* Marifrigoribacter gen. nov.

ma.ri.fri.go.ri.bac’ter L. neut. n. *mare,* the sea; L. neut. N. *frigor,* cold; N.L. masc. n. *bacter*, rod; N.L. masc. n. *Marifrigoribacter*, a rod from the cold of the sea.

## Data Availability

All data are publicly available at the NCBI under Bioproject PRJNA1129510. Individual genome accession and sequence read archive accession numbers are available in Table S2.

## SUPPLEMENTAL MATERIAL

Fig. S1 mbio.01555-25-s0001.eps SAR116 phylogeny.

Fig. S2 mbio.01555-25-s0002.eps Spatiotemporal ASV distribution.

Fig. S3 mbio.01555-25-s0003.eps ASV correlation.

Fig. S4 mbio.01555-25-s0004.eps ASV abundance by lineage.

Fig. S5 mbio.01555-25-s0005.eps ASV abundance by genome match.

Fig. S6 mbio.01555-25-s0006.eps Arctic SAR11 ITS phylogeny.

Fig. S7 mbio.01555-25-s0007.eps Genomic island gene cluster phylogeny.

Fig. S8 mbio.01555-25-s0008.eps Sequencing accuracy.

Supplemental Tables mbio.01555-25-s0009.xlsx Tables S1 to S6.

ASM does not own the copyrights to Supplemental Material that may be linked to, or accessed through, an article. The authors have granted ASM a non-exclusive, world-wide license to publish the Supplemental Material files. Please contact the corresponding author directly for reuse.
